# Increased Level of Angiopoietin Like Proteins 4 and 8 in People With Sleep Apnea

**DOI:** 10.3389/fendo.2018.00651

**Published:** 2018-11-13

**Authors:** Abdulmohsen Al-Terki, Mohamed Abu-Farha, Irina AlKhairi, Preethi T. Cherian, Devarajan Sriraman, Ambika Shyamsundar, Shamsha Ali, Fahd Almulla, Jaakko Tuomilehto, Jehad A. Abubaker

**Affiliations:** ^1^Otolaryngology, Head & Neck Surgery, Medical Division, Dasman Diabetes Institute, Kuwait City, Kuwait; ^2^Biochemistry and Molecular Biology Unit, Dasman Diabetes Institute, Kuwait City, Kuwait; ^3^National Dasman Diabetes Biobank, Dasman Diabetes Institute, Kuwait City, Kuwait; ^4^Research Division, Dasman Diabetes Institute, Kuwait City, Kuwait

**Keywords:** obstructive sleep apnea, ANGPTL4, ANGPTL8, apnea hypopnea index, polysomnography, lipid metabolism

## Abstract

**Objective:** Obstructive sleep apnea (OSA) is a sleep disorder caused by the complete or partial obstruction of the upper airways. The worldwide prevalence of OSA is increasing due to its close association with obesity epidemic and multiple health complications, such as hypertension, cardiovascular disease, and Type 2 diabetes. Angiopoietin-like protein (ANGPTL)-4 and ANGPTL8 (betatrophin) have been suggested to play a role in the development of these diseases through their role in regulating the metabolism of plasma lipid molecules. This study was designed to evaluate ANGPTL4 and 8 levels in an OSA group and a control group to clarify the effect of OSA on ANGPTL4 and 8 levels.

**Methods:** In total, 74 subjects were enrolled in this study, including 22 age- and body mass index (BMI)-matched controls with the Apnea Hypopnea Index (AHI) score of <5 events/h and 52 subjects with an AHI score of >5 events/h. Sleep apnea was assessed using a portable sleep test. ANGPTL4 and 8 levels were measured in plasma samples using enzyme-linked immunosorbent assay.

**Results:** Mean AHI score (2.5 ± 1.6) in the control group was significantly lower than that in the OSA group (22.9 ± 17.9; *p* < 0.0001). Leptin, interleukin-(IL) 6, insulin, and HOMA-IR values were higher in the OSA group than in the control group. ANGPTL8 level was higher in the OSA group (1130.0 ± 108.61 pg/mL) than in the control group (809.39 ± 108.78 pg/mL; *p* = 0.041). Similarly, ANGPTL4 was higher in the OSA group (179.26 ± 12.89 ng/mL) than in the control group (142.63 ±7.99 ng/mL; *p* = 0.018).

**Conclusion:** Our findings demonstrate that ANGPTL4 and 8 levels were increased in subjects with OSA, suggesting that the upregulation of these lipid metabolism regulators might play a role in lipid dysregulation observed in people with OSA.

## Background

Obstructive sleep apnea (OSA) is becoming increasingly prevalent worldwide due to widespread obesity, which is one of the main causes of OSA (1). OSA is caused by the partial or complete obstruction of the upper airways, which leads to the development of intermittent hypoxemia and hypercapnia as well as fragmented sleep and daytime lethargy. Presently, the diagnosis of OSA is established using polysomnography (PSG) and the Apnea Hypopnea Index (AHI) (1). OSA has been recognized as a major health problem associated with the increased risk of cardiovascular diseases (CVDs), hypertension, and Type 2 diabetes (T2D) (2–5). OSA has been shown to dysregulate the major pathways involved in the development of these diseases, such as inflammation, dyslipidemia, and insulin resistance (4, 6, 7).

Although the exact mechanism underlying OSA is unknown, inflammation and oxidative stress are considered to play important roles in the OSA development (8–14). Recent studies have shown that plasma levels of a wide range of proinflammatory mediators are elevated in patients with OSA (13, 15). One of the key hallmarks of OSA is intermittent hypoxia (IH) (10). IH has been implicated in inflammation, oxidative stress, and dyslipidemia (10, 16, 17). Recent studies have shown that OSA impairs the clearance of triglyceride-rich lipoproteins from human plasma, a process that is reversed using continuous positive airway pressure, which is a well-established treatment for OSA (18). The hydrolysis of triglyceride-rich lipoprotein into fatty acids is catalyzed by enzyme lipoprotein lipase (LPL), which is downregulated by hypoxia (19–21). LPL is involved in the hydrolysis of triglyceride from chylomicrons and very low-density lipoproteins (VLDLs) into fatty acids after food intake. LPL activity is regulated by various factors, including angiopoietin-like (ANGPTL) proteins, such as ANGPTL3, 4, and 8 (22–24). ANGPTLs are a family of proteins composed of eight members and have been associated with various metabolic pathways, including insulin resistance, oxidative stress, and dyslipidemia (24). ANGPTL4 and 8 are two members of this family and have been shown to play a role in lipid metabolism through regulating plasma lipid levels by inhibiting LPL activity (24–27). ANGPTL4 is a ubiquitously expressed protein that has been shown to be transcriptionally regulated by hypoxia (28–30). Moreover, ANGPTL8 has been shown to regulate the activity of LPL through its interaction with ANGPTL3 (31). Both our and other research groups have shown that ANGPTL8 is associated with insulin resistance and that its levels are increased in obesity, metabolic disease, and T2D (32–40). Considering that OSA is a known risk factor for dyslipidemia and the roles of ANGPTL4 and 8 in regulating the level of lipids in plasma as well as their involvement in metabolic-related pathways, we hypothesized that their levels would be dysregulated in OSA.

## Methods

### Study population and ethical statement

In total, 74 subjects were enrolled in this study, including 22 controls with an AHI score of <5 events/h and 52 subjects with an AHI score of >5 events/h. Sleep apnea was assessed using a portable sleep test. Control subjects were age- and body mass index (BMI)-matched. BMI was calculated using the standard BMI formula: body weight (kg)/height (m^2^). The study was approved by the ethical review board of the Dasman Diabetes Institute and conducted in accordance with the Declaration of Helsinki ethical guideline. Written informed consent was obtained from all subjects prior to participation in the study. Exclusion criteria were presence of CVDs and diabetes and a history of major illness.

### OSA assessment

PSG is known as the “gold standard” for the diagnosis of OSA. In our study, we used comprehensive portable PSG devices (“Type 2 devices”) to diagnose OSA. PSG devices monitor sleep stages, oxygen saturation, respiratory effort, heart rate and body position. These data are used to calculate AHI. AHI is the summation of apneas and hypopneas per hour of sleep, with “apnea” defined as the absence of airflow for ≥10 s and “hypopnea” as the reduction in respiratory effort with ≥4% oxygen desaturation. The “apnea index” (AI) is defined as the number events the of complete cessation of respiration for more than 10 s per hour, whereas the “hypopnea index” (HI) is defined as the number of events of partial airway obstructions per hour that result in 50% ventilation reduction, leading to arterial saturation of ≥ 4%. The subjects were requested to avoid caffeinated drinks and drugs that could affect their sleep. Polysomnographic monitoring was performed using the standard technique. OSA was diagnosed on the basis of the subject's AHI score, where an AHI of >5 events/h of sleep was considered abnormal, indicating that the subject presented with a sleep disease. An aberrant AHI score associated with enormous daytime sleepiness is the hallmark for OSA diagnosis.

### Blood collection and anthropometric and biochemical measurements

After each participant had signed the consent form, a fasting blood sample was collected in a vacutainer (EDTA tube). Plasma was prepared by the centrifugation of the blood containing tubes at 400 × g for 10 min and was then aliquoted and stored at −80°C until assayed (36, 41, 42). Blood pressure was measured using an Omron HEM-907XL digital sphygmomanometer. The mean blood pressure of three readings was recorded. Whole-body composition was determined using a dual-energy radiographic absorptiometry device (Lunar DPX, lunar radiation, Madison, WI). Fasting blood glucose (FBG), triglyceride (TG), total cholesterol, low-density lipoprotein (LDL), and high-density lipoprotein (HDL) were measured using the Siemens Dimension RXL chemistry analyzer (Diamond Diagnostics, Holliston, MA). Glycated hemoglobin was measured using the Variant^TM^ device (Bio-Rad, Hercules, CA). Insulin resistance was calculated using the HOMA-IR formula: FBG (mmol/L) × fasting insulin (mU/L)/22.5.

### Circulating biomarker levels

ANGPTL8 circulation level was measured using an enzyme-linked immunosorbent assay (ELISA) kit (Wuhan EIAAB Science Co, catalog number E1164H), following the method described in previous studies (36, 41, 42). Briefly, the plasma samples were thawed on ice and centrifuged at 10,000 × g for 5 min at 4°C to remove any cells or platelets remaining in the sample (36, 41, 42). No significant cross-reactivity with other proteins was observed. Intra-assay coefficients of variation were 3.1–5.7%, whereas inter-assay coefficients of variation were 6.2–9.8%. Plasma levels of ANGPTL4, leptin, and IL1, IL6, IL10, INF-γ, IP10, TNF-α were assessed using a multiplexing immunobead array platform according to the manufacturer's instructions (R & D Systems). Data were processed using the Bio-Plex Manager Software version 6 (Bio-Rad), with five-parametric curve fitting. To avoid batch-to-batch variations, samples were measured using reagents from the same batch.

### Statistical analysis

Student's *t*-test was used for comparisons between subjects with and without OSA. Spearman's correlation coefficients were used to determine associations between ANGPTL4 and 8 levels and OSA. All data were reported as mean ± standard deviation. Statistical assessments were two-sided and considered significant at *p* < 0.05. All analyses were performed using SAS (version 9r; SAS Institute).

## Results

### Study population characteristics

Our study sample comprised 74 subjects that were allocated into either a healthy control or OSA group on the basis of their AHI score (Table 1). The mean age for the control group was 39.6 ± 2.2 years, whereas that for the OSA group was 40.3 ± 1.6 years (*p* = 0.794). The mean BMI for the control group was 28.5 ± 0.9 kg/m^2^, whereas that for the OSA group was 29.5 ± 0.7 kg/m^2^ (*p* = 0.409). Overall, the OSA group showed slightly higher TG, LDL, and total cholesterol levels and lower HDL levels than the control group. However, these differences were not statistically significant.

**Table 1:** Characteristics of All subject included in this study according to their OSA state.

**Variables**	**Non-OSA**	**OSA**	***p*-value**
	**Average ± SE**	**Average ± SE**	
Age (Years)	39.6 ± 2.2	40.3 ± 1.6	0.794
BMI Kg/m2	28.5 ± 0.9	29.5 ± 0.7	0.409
Chol mmol/L	4.61 ± 0.20	4.99 ± 0.20	0.187
HDL mmol/L	1.16 ± 0.062	1.11 ± 0.063	0.584
LDL mmol/L	2.87 ± 0.18	3.31 ± 0.20	0.109
TGL mmol/L	1.12 ± 0.15	1.27 ± 0.10	0.426
GLU mmol/L	5.26 ± 0.098	5.22 ± 0.088	0.774
AI (Events/h)	0.8 ± 0.2	6.7 ± 2.3	**0.019**
HI (Events/h)	1.3 ± 0.3	14.0 ± 1.8	<**0.0001**
AHI (Events/h)	2.1 ± 0.3	20.8 ± 2.8	<**0.0001**
HOMAIR	3.30 ± 0.43	4.30 ± 0.40	0.095
Insulin U/L	13.94 ± 1.60	18.28 ± 1.61	0.063
Leptin ng/mL	5.97 ± 0.77	7.60 ± 0.63	0.097
IL6 pg/mL	13.7 ± 1.54	18.8 ± 2.16	0.057

*Statistically significant variables are in bold*.

### Polysomnographic data

AHI score was used for the diagnosis for OSA. AI, HI, and AHI data showed that the OSA group had a higher number of apnea and hypopnea events than the control group. Mean AI score for the OSA group was 6.7 ± 2.3 events/h, whereas that for the control group was 0.8 ± 0.2 events/h (*p* = 0.019). Mean HI score for the control group was 1.3 ± 0.3 events/h, whereas that for the OSA group was 14.0 ± 1.8 events/h (*p* < 0.0001). Finally, mean AHI score for the OSA group was 22.9 ± 17.9, whereas that for the control group was 2.5 ± 1.6 (*p* < 0.0001; Supplementary Figure 1).

### Plasma levels of inflammatory markers

**Figure 1:**
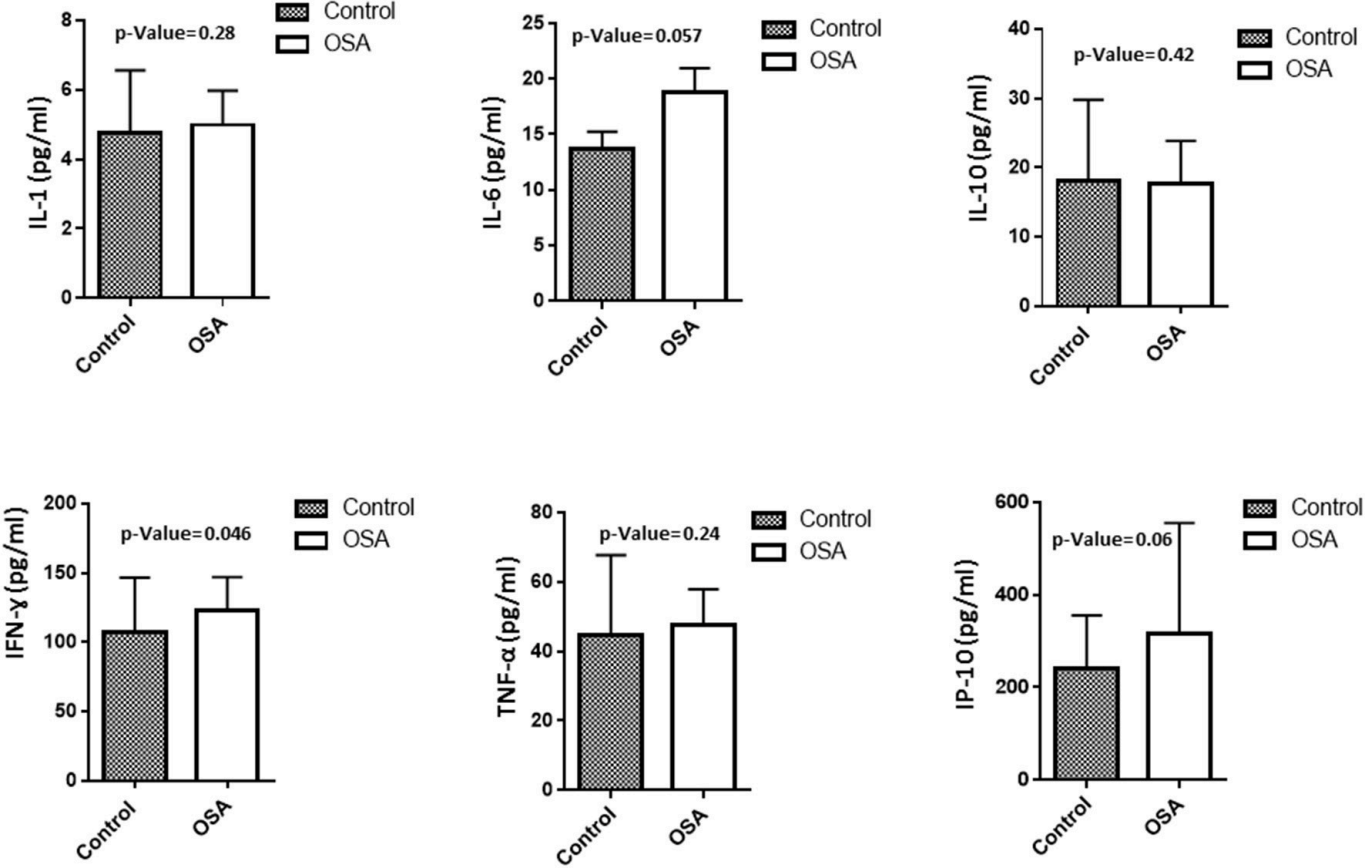
Plasma level of inflammatory related markers including IL1, IL6, IL10, INF-γ, IP10, TNF-α.

To assess level of inflammation within our cohort several markers have been measured including IL1, IL6, IL10, INF-γ, IP10, and TNF-α. Mean IL6 level in the OSA group was 18.8 ± 2.16 pg/mL, whereas that in the control group was 13.7 ± 1.54 pg/mL (*p* = 0.057). INF-γ and IP10 were also higher in people with OSA compared to their controls. INF-γ 123.23 ± 23.75 pg/mL vs. 107.50 ± 39.18 pg/mL (*p* = 0.046). On the other hand, the level of IP10 was 316.99 ± 237.89 pg/mL in people with OSA vs. 240.83 ± 114.75 pg/mL controls (*p* = 0.06). IL1, IL10, TNF-α did not change between people with OSA and their controls Figure 1.

### Plasma levels of insulin and leptin

**Figure 2:**
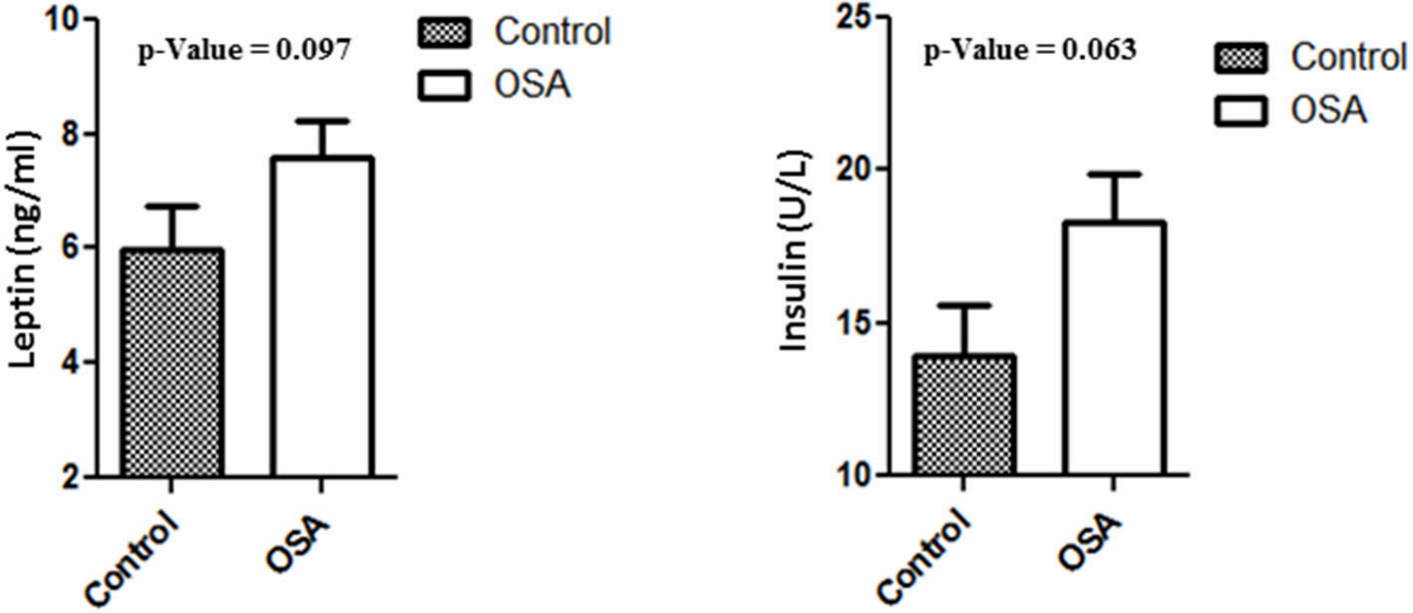
Increased plasma level of Leptin and Insulin in people with OSA compared to their age and BMI matched healthy controls.

Plasma levels of insulin, leptin, and IL6 were higher in the OSA group than in the control group. Even though the differences were not statistically significant, the values clearly showed an upward trend. Mean insulin level in the control group was 13.94 ± 1.60 U/L, whereas that in the OSA group was 18.28 ± 1.61 U/L (*p* = 0.063). Leptin and IL6 levels showed a trend similar to that of insulin level. Mean leptin level in the control group was 5.97 ± 0.77 ng/mL, whereas that in the OSA group was 7.60 ± 0.63 ng/mL (*p* = 0.097; Figure 2).

### Plasma levels of ANGPTL 4 and 8

**Figure 3:**
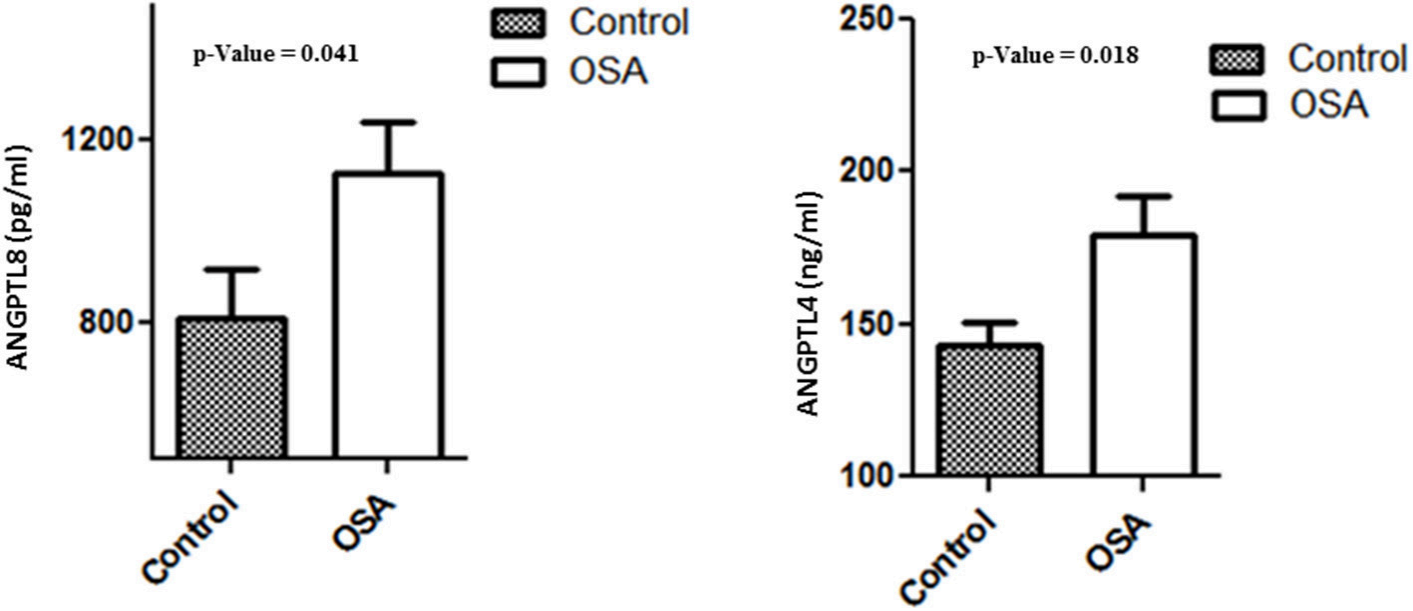
Plasma level of ANGPTL4 and 8 in people with OSA and their BMI and age matched healthy controls.

Plasma levels of both ANGPTL4 and 8 were significantly higher in the OSA group than in the control group. Mean ANGPTL4 level in the OSA group was 179.26 ± 12.89 ng/mL, whereas that in the control group was and 142.63 ± 7.99 ng/mL (*p* = 0.018). Mean ANGPTL8 level in the control group was 809.39 ± 108.78 pg/mL, whereas that in the OSA group was 1130.00 ± 108.61 pg/mL (*p* = 0.041; Figure 3).

## Discussion

Roles of ANGPTL3, 4, and 8 in dyslipidaemia, obesity, and diabetes, diseases that are closely associated with OSA, are well-established. Our analysis of ANGPTL4 and 8 levels in subjects with OSA and healthy controls has shown that levels of both proteins were significantly increased in subjects with OSA. Plasma level of other inflammatory proteins (IL6), adipokines (leptin), and insulin were also higher in subjects with OSA although these differences were not statistically significant. Plasma lipid levels in the OSA group were also slightly high, but these differences were not statistically significant. Taken together, findings of this study highlight the potential role of ANGPTL 4 and 8 in OSA, and as such, they could act as early markers of OSA. The dysregulation of ANGPTL 4 and 8 might be involved in the development of diseases, such as dyslipidaemia, possibly through hypoxia.

The prevalence of OSA is increasing worldwide due to the similarly increasing prevalence of obesity. A recent meta-analysis has indicated that 20% of adults suffer from moderate OSA and over 7% of adults suffer from severe OSA (1). Growing evidence has suggested the role of OSA in the development of hypertension, stroke, T2D, and dyslipidaemia (2, 3, 11, 12). IH is one of the hallmarks of OSA and is caused by the partial or complete obstruction of the upper airways (1, 10). Many studies have reported that the cause underlying the increased risks associated with OSA is induced IH and its associated pathological processes, such as oxidative stress, insulin resistance, and dyslipidaemia (2, 3, 11, 12). IH has also been shown to play an important role in inhibiting LPL activity and increasing ANGPTL4 levels, leading to dyslipidaemia. Our data show that ANGPTL4 level was increased in subjects with OSA, which highlights the potential role of ANGPTL4 in OSA and the associated complications, such as dyslipidaemia. ANGPTL4 has been shown to be increased in subjects with OSA, and it has been suggested that this increase is mediated by hypoxia. Additionally, our data confirm the assumption that the inhibition of ANGPTL4 is responsible for decreasing VLDL to normal levels and that this reduction in VLDL level is caused by increased LPL activity that follows ANGPTL4 inhibition. In addition, our data confirms for the first time that ANGPTL8 might be increased in OSA further inhibiting LPL activity.

ANGPTL8 is another member of the ANGPTL family that lacks fibrinogen-like domain (31, 43–45). Some members of this family have been shown to regulate glucose, lipid, and energy metabolisms (27). In particular, ANGPTL3 and 4 have been shown to regulate lipid metabolism through their regulation of the rate limiting enzyme in LPL hydrolysis (27). ANGPTL8 has also been shown to play a similar role in regulating LPL through its interaction with ANGPTL3 (31). The role of ANGPTL4 in regulating plasma lipid levels has been under investigation as a therapeutic target (46, 47). Many studies have shown that the loss-of-function mutations in ANGPTL4, particularly the E40K substitution, are associated with reduced plasma levels of TG and HDL (26, 48–50). Further, recent data have demonstrated that carriers of the E40K substitution have a significantly lower risk of developing coronary artery disease than non-carriers (48). Furthermore, it has been shown that mice injected with ANGPTL4 monoclonal antibodies exhibit a low plasma TG as well as increased LPL activity (27). Taken together, these findings suggest that increased ANGPTL4 levels in our study population support the role of ANGPTL4 in OSA development in this population. A possible mechanism underlying this could be ANGPTL4 activation by hypoxia, a known inducer of ANGPTL4 expression (29, 51). It's possible that the increased ANGPTL4 and possibly 8 level will lead to increased expression of inflammatory markers. In this study we have observed increased expression of IL6, INF-γ, and IP10 which have shown to be downregulated in ANGPTL4 adipose tissue specific knockout mice model (52). Nonetheless, ANGPTL4 has also been suggested to decrease inflammation that is caused by the increased plasma TG level associated with ANGPTL4 inhibition of LPL activity and fatty acid uptake (53). Weather ANGPTL4 plays a pro-or anti-inflammatory role in this case will require further studies.

ANGPTL8 or betatrophin has been recognized as a dual-role protein involved in regulating lipid metabolism through its role in regulating LPL. Our and other research groups have recently shown that ANGPTL8 levels is increased in T2D as well as in obesity, which suggests the pathogenic role of this protein in these diseases (33, 34). Our earlier results have indicated that plasma ANGPTL8 level is positively correlated with BMI, SBP, HOMA-IR, and metabolic syndrome (MetS) (33, 34, 36, 54, 55). This indicates that ANGPTL8 may be involved in the mechanism of obesity and MetS. Obesity and MetS are closely associated with OSA. Obesity, particularly central adiposity, is a key risk factor for OSA. Moreover, IH is associated with inflammation, oxidative stress, and dyslipidemia. A recent report has shown that OSA reduced the clearance of triglyceride-rich lipoproteins from human plasma through the inhibition of LPL (1, 9, 10, 29). Moreover, our current findings suggest that hypoxia is a major inducer of ANGPTL8 expression, which could explain the upregulation of ANGPTL8 in subjects with OSA. These findings indicate that ANGPTL8 may be involved in the crosstalk between proteins regulating obesity, hypoxia, and OSA. Additionally, ANGPTL8 can be also playing a role in inducing inflammation. We have previously showed that ANGPTl8 was positively associated with HsCRP (54). Additionally, ANGPTL8 has been suggested to be induced by multiple inflammatory to regulate proinflammatory reactions such as the inhibition of the TNFα-induced NF-κB activation (56). Combined with our observation regarding the level of ANGPTL4 further studies are required to establish the role of these biomarkers in OSA and their involvement in regulating immune response. Given the cross-sectional nature of our study, this constitute one of the main limitation of the current study that does not allow us to establish the causality and the role of ANGPTL4 and 8 proteins in the development of OSA complications.

## Conclusions

In conclusion, this study demonstrates that plasma levels of ANGPTL4 and 8 were increased in subjects with OSA compared with those in healthy controls. Hypoxia and obesity may be the major mechanisms underlying the upregulation of these lipid metabolism regulators in subjects with OSA that can also increase inflammation status within these individuals. Further, our data indicate that increased plasma ANGPTL8 level may be an independent predictor of the presence of OSA.

## Availability of data and material

Data will only be shared upon request from the corresponding authors due to unpublished data and ethical restriction by the institute.

## Author contributions

AA-T: study design and sleep study data interpretation; MA-F: study design, data analysis wrote the manuscript and directed laboratory investigation; IA and PC: performing ELISA assay and critical revision of manuscript; DS: analyzing and managing data; AS and SA: sample handling and storage; FA: interpreting data and critical revision of the manuscript; JT: critical revision of the manuscript; JA: designing the study, interpreting data and critically revising the manuscript.

### Conflict of interest statement

The authors declare that the research was conducted in the absence of any commercial or financial relationships that could be construed as a potential conflict of interest.

## Supplementary material

Supplementary Figure 1 Polysomnographic data showing the Apnea index (AI) as well as the hypopnea index (HI) as well as the combined AI and HI index the AHI for people with or without OSA. OSA was diagnosed based on an AHI >5 events/h. ^*^*P* < 0.05 as determined using student's *t*-test.

## Abbreviations

AI: Apnea Index
HI: Hypoapnea Index
AHI: Apnea/hypopnea index
BMI: Body mass index
CV: Coefficients of variation
HDL: High-density lipoprotein
LDL: Low-density lipoprotein
ELISA: Enzyme-linked immunosorbent assay
TC: Total cholesterol
FBG: Fasting blood glucose
HbA1C: Glycated hemoglobin
ANGPTL: Angiopoietin-like protein
SBP: Systolic blood pressure
DBP: Diastolic blood pressure
TG: Triglyceride
IH: Intermittent hypoxia.

## References

[1] Levy P Kohler M McNicholas WT Barbe F McEvoy RD Somers VK. Obstructive sleep apnoea syndrome. Nat Rev Dis Primers (2015) 1:15015. 10.1038/nrdp.2015.15 27188535

[2] Gopalakrishnan P Tak T. Obstructive sleep apnea and cardiovascular disease. Cardiol Rev. (2011) 19:279–90. 10.1097/CRD.0b013e318223bd08 21983316

[3] Kendzerska T Gershon AS Hawker G Tomlinson G Leung RS. Obstructive sleep apnea and incident diabetes. A historical cohort study. Am J Respir Crit Care Med. (2014) 190:218–25. 10.1164/rccm.201312-2209OC 24897551

[4] Rasche K Keller T Tautz B Hader C Hergenc G Antosiewicz J. Obstructive sleep apnea and type 2 diabetes. Eur J Med Res. (2010) 15(Suppl. 2):152–6. 10.3389/fneur.2012.00126 21147644 PMC4360282

[5] Godoy J Mellado P Tapia J Santin J. Obstructive sleep apnea as an independent stroke risk factor: possible mechanisms. Curr Mol Med. (2009) 9:203–9. 10.2174/156652409787581556 19275628

[6] Clarenbach CF West SD Kohler M. Is obstructive sleep apnea a risk factor for diabetes? Discov Med. (2011) 12:17–24. 21794205

[7] Fredheim JM Rollheim J Omland T Hofso D Roislien J Vegsgaard K. Type 2 diabetes and pre-diabetes are associated with obstructive sleep apnea in extremely obese subjects: a cross-sectional study. Cardiovasc Diabetol. (2011) 10:84. 10.1186/1475-2840-10-84 21943153 PMC3206416

[8] Ryan S. Adipose tissue inflammation by intermittent hypoxia: mechanistic link between obstructive sleep apnoea and metabolic dysfunction. J Physiol. (2017) 595:2423–30. 10.1113/JP273312 27901270 PMC5390885

[9] Passali D Corallo G Yaremchuk S Longini M Proietti F Passali GC. Oxidative stress in patients with obstructive sleep apnoea syndrome. Acta Otorhinolaryngol Ital. (2015) 35:420–5. 10.14639/0392-100X-895. 26900248 PMC4755047

[10] Toraldo DM DE Nuccio F DE Benedetto M Scoditti E. Obstructive sleep apnoea syndrome: a new paradigm by chronic nocturnal intermittent hypoxia and sleep disruption. Acta Otorhinolaryngol Ital. (2015) 35:69–74. 26019388 PMC4443563

[11] Sanchez-de-la-Torre M. Campos-Rodriguez F Barbe F Obstructive sleep apnoea and cardiovascular disease. Lancet Respir Med. (2013) 1:61–72. 10.1016/S2213-2600(12)70051-6 24321805

[12] Lam JC Mak JC Ip MS. Obesity, obstructive sleep apnoea and metabolic syndrome. Respirology (2012) 17:223–36. 10.1111/j.1440-1843.2011.02081.x 21992649

[13] Kimoff RJ Hamid Q Divangahi M Hussain S Bao W Naor N. Increased upper airway cytokines and oxidative stress in severe obstructive sleep apnoea. Eur Respir J. (2011) 38:89–97. 10.1183/09031936.00048610 20847078

[14] Bradley TD Floras JS. Obstructive sleep apnoea and its cardiovascular consequences. Lancet (2009) 373:82–93. 10.1016/S0140-6736(08)61622-0 19101028

[15] Bielicki P MacLeod AK Douglas NJ Riha RL. Cytokine gene polymorphisms in obstructive sleep apnoea/hypopnoea syndrome. Sleep Med. (2015) 16:792–5. 10.1016/j.sleep.2015.01.006 25953302

[16] Wenger RH. Cellular adaptation to hypoxia: O2-sensing protein hydroxylases, hypoxia-inducible transcription factors, and O2-regulated gene expression. FASEB J. (2002) 16:1151–62. 10.1096/fj.01-0944rev 12153983

[17] Park JW Chun YS Kim MS. Hypoxia-inducible factor 1-related diseases and prospective therapeutic tools. J Pharmacol Sci. (2004) 94:221–32. 10.1254/jphs.94.221 15037806

[18] Lin MT Lin HH Lee PL Weng PH Lee CC Lai TC. Beneficial effect of continuous positive airway pressure on lipid profiles in obstructive sleep apnea: a meta-analysis. Sleep Breath (2015) 19:809–17. 10.1007/s11325-014-1082-x 25450153 PMC4559086

[19] Yao Q Shin MK Jun JC Hernandez KL Aggarwal NR Mock JR. Effect of chronic intermittent hypoxia on triglyceride uptake in different tissues. J Lipid Res. (2013) 54:1058–65. 10.1194/jlr.M034272 23386706 PMC3605982

[20] Jun JC Shin MK Yao Q Bevans-Fonti S Poole J. Acute hypoxia induces hypertriglyceridemia by decreasing plasma triglyceride clearance in mice. Am J Physiol Endocrinol Metab. (2012) 303:E377–88. 10.1152/ajpendo.00641.2011 22621867 PMC3423119

[21] Drager LF Li J Shin MK Reinke C Aggarwal NR Jun JC. Intermittent hypoxia inhibits clearance of triglyceride-rich lipoproteins and inactivates adipose lipoprotein lipase in a mouse model of sleep apnoea. Eur Heart J. (2012) 33:783–90. 10.1093/eurheartj/ehr097 21478490 PMC3303712

[22] Haller JF Mintah IJ Shihanian LM Stevis P Buckler D Alexa-Braun CA. ANGPTL8 requires ANGPTL3 to inhibit lipoprotein lipase and plasma triglyceride clearance. J Lipid Res. (2017) 58:1166–73. 10.1194/jlr.M075689 28413163 PMC5454515

[23] Reimund M Kovrov O Olivecrona G Lookene A. Lipoprotein lipase activity and interactions studied in human plasma by isothermal titration calorimetry. J Lipid Res. (2017) 58:279–88. 10.1194/jlr.D071787 27845686 PMC5234706

[24] Zhang R. The ANGPTL3-4-8 model, a molecular mechanism for triglyceride trafficking. Open Biol. (2016) 6:150272. 10.1098/rsob.150272 27053679 PMC4852456

[25] Abu-Farha M Melhem M Abubaker J Behbehani K Alsmadi O Elkum N. ANGPTL8/Betatrophin R59W variant is associated with higher glucose level in non-diabetic Arabs living in Kuwaits. Lipids Health Dis. (2016) 15:26. 10.1186/s12944-016-0195-6 26864934 PMC4750355

[26] Abid K Trimeche T Mili D Msolli MA Trabelsi I Nouira S Kenani A. ANGPTL4 variants E40K and T266M are associated with lower fasting triglyceride levels and predicts cardiovascular disease risk in Type 2 diabetic Tunisian population. Lipids Health Dis. (2016) 15:63. 10.1186/s12944-016-0231-6 27004807 PMC4804568

[27] Santulli G. Angiopoietin-like proteins: a comprehensive look. Front Endocrinol. (2014) 5:4. 10.3389/fendo.2014.00004 24478758 PMC3899539

[28] Hata S Nomura T Iwasaki K Sato R Yamasaki M Sato F. Hypoxia-induced angiopoietin-like protein 4 as a clinical biomarker and treatment target for human prostate cancer. Oncol Rep. (2017) 38:120–8. 10.3892/or.2017.5669 28560449

[29] Hu K Babapoor-Farrokhran S Rodrigues M Deshpande M Puchner B Kashiwabuchi F. Hypoxia-inducible factor 1 upregulation of both VEGF and ANGPTL4 is required to promote the angiogenic phenotype in uveal melanoma. Oncotarget (2016) 7:7816–28. 10.18632/oncotarget.6868 26761211 PMC4884956

[30] Babapoor-Farrokhran S Jee K Puchner B Hassan SJ Xin X Rodrigues M. Angiopoietin-like 4 is a potent angiogenic factor and a novel therapeutic target for patients with proliferative diabetic retinopathy. Proc Natl Acad Sci USA. (2015) 112:E3030–9. 10.1073/pnas.1423765112 26039997 PMC4466723

[31] Quagliarini F Wang Y Kozlitina J Grishin NV Hyde R Boerwinkle E. Atypical angiopoietin-like protein that regulates ANGPTL3. Proc Natl Acad Sci USA. (2012) 109:19751–6. 10.1073/pnas.1217552109 23150577 PMC3511699

[32] Abu-Farha M Abubaker J Tuomilehto J. ANGPTL8 (betatrophin) role in diabetes and metabolic diseases. Diabetes Metab Res Rev. (2017) 33:e2919. 10.1002/dmrr.2919 28722798

[33] Abu-Farha M Sriraman D Cherian P AlKhairi I Elkum N Behbehani K. Circulating ANGPTL8/Betatrophin is increased in obesity and reduced after exercise training. PLoS ONE (2016) 11:e0147367. 10.1371/journal.pone.0147367 26784326 PMC4718617

[34] Abu-Farha M Al-Khairi I Cherian P Chandy B Sriraman D Alhubail A. Increased ANGPTL**3**:4 and ANGPTL8/betatrophin expression levels in obesity and T2D. Lipids Health Dis. (2016) 15:181. 10.1186/s12944-016-0337-x 27733177 PMC5062897

[35] Yamada H Saito T Aoki A Asano T Yoshida M Ikoma A. Circulating betatrophin is elevated in patients with type 1 and type 2 diabetes. Endocr J. (2015) 62:417–21. 10.1507/endocrj.EJ14-0525 25753914

[36] Abu-Farha M Abubaker J Al-Khairi I Cherian P Noronha F Hu FB Higher plasma betatrophin/ANGPTL8 level in type 2 diabetes subjects does not correlate with blood glucose or insulin resistance. Sci Rep. (2015) 5:10949 10.1038/srep10949 26077345 PMC4650613

[37] Zhang R Abou-Samra AB. A dual role of lipasin (betatrophin) in lipid metabolism and glucose homeostasis: consensus and controversy. Cardiovasc Diabetol. (2014) 13:133. 10.1186/s12933-014-0133-8 25212743 PMC4172915

[38] Hu H Sun W Yu S Hong X Qian W Tang B. Increased circulating levels of betatrophin in newly diagnosed type 2 diabetic patients. Diabetes Care (2014) 37:2718–22. 10.2337/dc14-0602 25024395

[39] Fu Z Berhane F Fite A Seyoum B Abou-Samra AB Zhang R. Elevated circulating lipasin/betatrophin in human type 2 diabetes and obesity. Sci Rep. (2014) 4:5013. 10.1038/srep05013 24852694 PMC5381405

[40] Fenzl A Itariu BK Kosi L Fritzer-Szekeres M Kautzky-Willer A Stulnig TM Circulating betatrophin correlates with atherogenic lipid profiles but not with glucose and insulin levels in insulin-resistant individuals. Diabetologia (2014) 57:1204–8. 10.1007/s00125-014-3208-x 24623100

[41] Abubaker J Tiss A Abu-Farha M Al-Ghimlas F Al-Khairi I Baturcam E. DNAJB3/HSP-40 cochaperone is downregulated in obese humans and is restored by physical exercise. PLoS ONE (2013) 8:e69217. 10.1371/journal.pone.0069217 23894433 PMC3722167

[42] Abu-Farha M Cherian P Al-Khairi I Tiss A Khadir A Kavalakatt S. DNAJB3/HSP-40 cochaperone improves insulin signaling and enhances glucose uptake *in vitro* through JNK repression. Sci Rep. (2015) 5:14448. 10.1038/srep14448 26400768 PMC4585859

[43] Ren G Kim JY Smas CM. Identification of RIFL, a novel adipocyte-enriched insulin target gene with a role in lipid metabolism. Am J Physiol Endocrinol Metab. (2012) 303:E334–51. 10.1152/ajpendo.00084.2012 22569073 PMC3423120

[44] Zhang R. Lipasin, a novel nutritionally-regulated liver-enriched factor that regulates serum triglyceride levels. Biochem Biophys Res Commun. (2012) 424:786–92. 10.1016/j.bbrc.2012.07.038 22809513

[45] Fu Z Yao F Abou-Samra AB Zhang R. Lipasin, thermoregulated in brown fat, is a novel but atypical member of the angiopoietin-like protein family. Biochem Biophys Res Commun. (2013) 430:1126–31. 10.1016/j.bbrc.2012.12.025 23261442

[46] Kersten S. Physiological regulation of lipoprotein lipase. Biochim Biophys Acta (2014) 1841:919–33. 10.1016/j.bbalip.2014.03.013 24721265

[47] Dijk W Kersten S. Regulation of lipoprotein lipase by Angptl4. Trends Endocrinol Metab. (2014) 25:146–55. 10.1016/j.tem.2013.12.005 24397894

[48] Myocardial Infarction G.C.A. Investigators EC Stitziel NO Stirrups KE Masca NG Erdmann J Coding variation in ANGPTL4, LPL, and SVEP1 and the risk of coronary disease. N Engl J Med. (2016) 374:1134–44. 10.1056/NEJMoa1507652 26934567 PMC4850838

[49] Dewey FE Gusarova V O'Dushlaine C Gottesman O Trejos J Hunt C. Inactivating variants in ANGPTL4 and Risk of coronary artery disease. N Engl J Med. (2016) 374:1123–33. 10.1056/NEJMoa1510926 26933753 PMC4900689

[50] Romeo S Pennacchio LA Fu Y Boerwinkle E Tybjaerg-Hansen A Hobbs HH. Population-based resequencing of ANGPTL4 uncovers variations that reduce triglycerides and increase HDL. Nat Genet. (2007) 39:513–6. 10.1038/ng1984 17322881 PMC2762948

[51] Gonzalez-Muniesa P de Oliveira C Perez de Heredia F Thompson MP Trayhurn P Fatty acids and hypoxia stimulate the expression and secretion of the adipokine ANGPTL4 (angiopoietin-like protein 4/ fasting-induced adipose factor) by human adipocytes. J Nutrigenet Nutrigenom. (2011) 4:146–53. 10.1159/000327774 21709421

[52] Aryal B Singh AK Zhang X Varela L Rotllan N Goedeke L. Absence of ANGPTL4 in adipose tissue improves glucose tolerance and attenuates atherogenesis. JCI Insight (2018) 3:97918. 10.1172/jci.insight.97918 29563332 PMC5926923

[53] Lichtenstein L Mattijssen F de Wit NJ Georgiadi A Hooiveld GJ van der Meer R. Angptl4 protects against severe proinflammatory effects of saturated fat by inhibiting fatty acid uptake into mesenteric lymph node macrophages. Cell Metab. (2010) 12:580–92. 10.1016/j.cmet.2010.11.002 21109191 PMC3387545

[54] Abu-Farha M Abubaker J Al-Khairi I Cherian P Noronha F Kavalakatt S. Circulating angiopoietin-like protein 8 (betatrophin) association with HsCRP and metabolic syndrome. Cardiovasc Diabetol. (2016) 15:25. 10.1186/s12933-016-0346-0 26850725 PMC4743238

[55] Abu-Farha M Abubaker J Noronha F Al-Khairi I Cherian P Alarouj M. Lack of associations between betatrophin/ANGPTL8 level and C-peptide in type 2 diabetic subjects. Cardiovasc Diabetol. (2015) 14:112. 10.1186/s12933-015-0277-1 26289721 PMC4546083

[56] Zhang Y Guo X Yan W Chen Y Ke M Cheng C. ANGPTL8 negatively regulates NF-κB activation by facilitating selective autophagic degradation of IKKγ. Nat Commun. (2017) 8:2164. 10.1038/s41467-017-02355-w 29255244 PMC5735157

