# Supplementary figures and images for: Increased Level of Angiopoietin Like Proteins 4 and 8 in People With Sleep Apnea
Source: Front Endocrinol (Lausanne). 2018 Nov 13;9:651. doi: 10.3389/fendo.2018.00651 (PMC6262344; doi:10.3389/fendo.2018.00651)

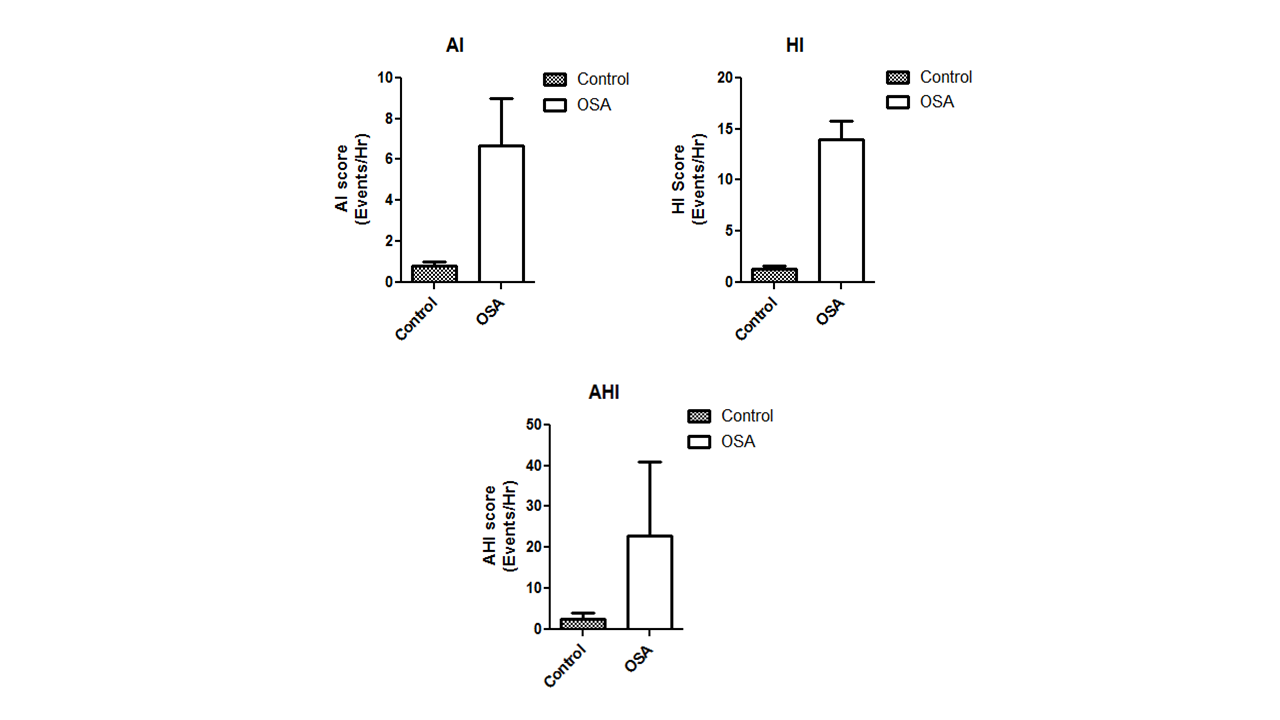

Supplement: Supplementary Figure 1 — Polysomnographic data showing the Apnea index (AI) as well as the hypopnea index (HI) as well as the combined AI and HI index the AHI for people with or without OSA. OSA was diagnosed based on an AHI >5 events/h. *P < 0.05 as determined using student's t-test. [file Image_1.TIF]
